# Evaluation of *Salmonella* Typhimurium Lacking *fruR*, *ssrAB*, or *hfq* as a Prophylactic Vaccine against *Salmonella* Lethal Infection

**DOI:** 10.3390/vaccines10091413

**Published:** 2022-08-28

**Authors:** Soyeon Park, Bogyo Jung, Eunsuk Kim, Hyunjin Yoon, Tae-Wook Hahn

**Affiliations:** 1 Department of Veterinary Medicine and Institute of Veterinary Science, Kangwon National University, Chuncheon 24341, Korea; 2 Department of Molecular Science and Technology, Ajou University, Suwon 16499, Korea

**Keywords:** *Salmonella* vaccine, *Salmonella* infection, *fruR*, *cra*

## Abstract

Non-typhoidal *Salmonella* (NTS) is one of the primary causes of foodborne gastroenteritis; occasionally, it causes invasive infection in humans. Because of its broad host range, covering diverse livestock species, foods of animal origin pose a critical threat of NTS contamination. However, there is currently no licensed vaccine against NTS infection. FruR, also known as Cra (catabolite repressor/activator), was initially identified as the transcriptional repressor of the fructose (*fru*) operon, and then found to activate or repress the transcription of many different genes associated with carbon and energy metabolism. In view of its role as a global regulator, we constructed a live attenuated vaccine candidate, Δ*fruR*, and evaluated its prophylactic effect against NTS infection in mice. A *Salmonella* Typhimurium mutant strain lacking *fruR* was defective in survival inside macrophages and exhibited attenuated virulence in infected mice. Immunization with the Δ*fruR* mutant stimulated the production of antibodies, including the IgG, IgM, and IgG subclasses, and afforded a protection of 100% to mice against the challenge of lethal infection with a virulent *Salmonella* strain. The prophylactic effect obtained after Δ*fruR* immunization was also validated by the absence of signs of hepatosplenomegaly, as these mice had comparable liver and spleen weights in comparison with healthy mice. These results suggest that the Δ*fruR* mutant strain can be further exploited as a promising vaccine candidate against *Salmonella* lethal infection.

## 1. Introduction

Salmonellosis caused by non-typhoidal *Salmonella* (NTS) is a major foodborne gastrointestinal disease worldwide [1,2]. The Centers for Disease Control and Prevention reported that *Salmonella enterica* is responsible for over 1 million cases of foodborne disease per year [3]. The symptoms of NTS infection, including diarrhea, abdominal cramps, and fever, are generally self-limited in healthy individuals. However, some NTS strains are invasive and enter the bloodstream, causing systemic infections such as septicemia, meningitis, and hepatosplenomegaly [4,5]. Infection with invasive NTS is often lethal in the old and the weak, especially immunocompromised patients. Furthermore, considering the increased prevalence of multiple-drug-resistant NTS strains, invasive NTS poses a serious threat to human health [6,7]. However, despite the maleficence of invasive NTS, there is no available licensed vaccine for the prevention of invasive NTS infection [8].

In an effort to develop vaccines against invasive NTS, various types of vaccines, including attenuated vaccines, whole-cell killed vaccines, and subunit vaccines, have been evaluated [9]. Among them, attenuated vaccines have been extensively explored because of their cost-effectiveness and outstanding prophylactic effects against infectious agents. A successful attenuated vaccine should be strongly attenuated regarding virulence, but stimulate a significant humoral and cellular immunity in the host after inoculation. There have been several reports about attenuated vaccines against NTS infections [9,10,11]. However, these attenuated vaccines are not licensed for humans.

The aim of this study is to develop a live attenuated vaccine against NTS infection. For the first step towards successful vaccine development, we evaluated the potency of *Salmonella* strains lacking FruR, SsrAB, or Hfq as live attenuated vaccines. SsrAB is a two-component regulatory system of the *Salmonella* pathogenicity island-2 (SPI-2), which is a critical virulence determinant that is required for *Salmonella* survival and replication inside host cells [12,13]. Hfq was originally identified as host factor I for bacteriophage Q beta replication, and then found to regulate the translation of more than 20% of all *Salmonella* proteins as a post-transcriptional regulator [14,15]. FruR, in addition to repressing the fructose (*fru*) operon transcriptionally, is extensively involved in carbon utilization and energy metabolism [16], including glycolysis, the Krebs cycle, the glyoxylate shunt, gluconeogenesis, electron transport, and pentose phosphate pathways [17]. Moreover, *Salmonella* lacking FruR is attenuated regarding its virulence in animal infection models [18]. Regarding the multifaceted roles of FruR in bacterial virulence regulation, as well as metabolic regulation, *Salmonella* lacking FruR is an ideal candidate live attenuated vaccine against NTS infection. Here, we evaluated the prophylactic effects of three *Salmonella enterica* serovar Typhimurium strains lacking FruR, SsrAB, or Hfq against invasive NTS infection. Among these three strains, *Salmonella* devoid of FruR exhibited significantly decreased virulence accompanied by strong immunogenicity in a mouse model, protecting host animals against NTS challenges. These results suggest that the Δ*fruR S.* Typhimurium strain is an ideal attenuated vaccine candidate for preventing invasive NTS infections.

## 2. Materials and Methods

### 2.1. Bacterial Strains, Media and Culture Conditions

All bacterial strains used in this study are listed in Supplementary Table S1. *Salmonella enterica* serovar Typhimurium (ST) 1120, which is an isolate from swine feces in Korea [19], was used as the parent strain for the generation of the Δ*fruR*, Δ*ssrAB*, and Δ*hfq* mutant strains. *Salmonella* strains lacking *fruR, ssrAB*, and *hfq* were originally constructed in ST14028, as described in a previous study [20], and the mutated genetic alleles marked with a kanamycin resistance cassette were transferred to ST1120 using P22 HT105/1 int-201-mediated transduction [21]. The antibiotic cassette was subsequently removed by introducing pCP20, which produces a flip recombinase [20,22]. The removal of the kanamycin resistance cassette was examined by incubating the recombinants on an agar plate containing kanamycin at 50 μg/mL. The final non-polar deletions without the antibiotic cassette were verified using diagnostic PCR. Primers used in the diagnostic PCR are listed in Table S2. ST2173 used in the challenge experiments was constructed by transforming ST14028 with the pBBR1-MCS4 plasmid as described previously [23]. *Salmonella* strains were cultured in Luria–Bertani (LB) medium at 37 °C with antibiotics when required, unless otherwise specified. All antibiotics were purchased from Sigma-Aldrich (St. Louis, MO, USA). The biochemical characteristics of bacteria were identified using the analytical profile index (API) 20E kit (bioMérieux, Inc., Durham, NC, USA).

### 2.2. Intracellular Survival Assay

*Salmonella* survival inside macrophages was assayed as described in previous studies [19,23]. Briefly, murine macrophage RAW264.7 cells were cultured in Dulbecco’s Modified Eagle’s Medium (DMEM) supplemented with 10% fetal bovine serum and seeded in 24-well culture plates at a cell density of 2 × 10^5^ cells/well, respectively. Macrophage cells were infected with *Salmonella* cells of wild-type (WT) and mutant strains at a multiplicity of infection (MOI) of 100. All bacterial strains were cultured in LB broth overnight prior to RAW264.7 infection. Thirty minutes after bacterial infection, the macrophage cells were washed with phosphate-buffered saline (PBS) and replenished with fresh DMEM containing gentamicin (100 μg/mL), to remove extracellular bacteria. At 1.5 h of incubation with 100 μg/mL gentamicin, half of the infected cells were lysed using 1% Triton X-100 and the lysates were plated on LB agar to estimate the number of phagocytosed bacteria. The remainder of the cells were washed with PBS and further incubated in fresh DMEM containing 10 μg/mL gentamicin for an additional 8 h. To compare intracellular survival abilities among the bacterial strains, the RAW264.7 cells infected with *Salmonella* for 10 h in total were lysed, and the lysates were spread on agar plates as described above. *Salmonella* survival assay was conducted in triplicate and the average values were plotted. 

### 2.3. Motility Assays

Bacterial swimming motility was tested on semi-solid agar plates (0.3% agar) as described previously [24]. Briefly, bacterial cells were cultured to an optical density at 600 nm (OD_600_) of 0.6–0.7 and were spotted onto semi-solid agar with a needle. The agar plates were incubated at 37 °C. The diameters of the bacterial migration zones were measured at 0.5, 2, 4, 6, 8, and 12 h after spotting, respectively. Bacterial motility assay was performed in triplicate and the diameters were averaged at each time point. 

### 2.4. Analysis of Outer Membrane Proteins (OMPs)

Bacterial OMPs were isolated according to the method described by Barenkamp et al. [25]. Bacterial cells grown in LB broth were harvested by centrifugation at 2500× *g* for 20 min and resuspended in 10 mM HEPES (*N*-2-hydroxyethylpiperazine *N*′-2-ethanesulfonic acid) buffer. Bacterial cells were mechanically disrupted using a sonicator (Vibra-cell ultrasonic liquid processors; Sonics & Materials Inc., Newtown, CT, USA) and subjected to ultracentrifugation (45 Ti rotor; Beckman Coulter, Brea, CA, USA) at 100,000 × *g* at 4 °C for 1 h to collect the membrane fraction as a pellet. The membrane fraction was resuspended in 1% (wt/vol) *N*-lauryl sarcosine (Sarkosyl, Sigma-Aldrich, St. Louis, MO, USA) in 10% HEPES to solubilize inner membrane proteins, and processed using ultracentrifugation at 100,000 × *g* at 4 °C for 1 h. The resultant pellet containing OMPs was resuspended in PBS and the protein concentration was measured using the Pierce BCA protein assay kit (Thermo Scientific Inc., Waltham, MA, USA). OMP samples equivalent to 10 μg were loaded and separated in 12% sodium dodecyl sulfate polyacrylamide gel electrophoresis (SDS–PAGE), followed by staining of the gels with Coomassie Brilliant Blue, to visualize OMP profiles.

### 2.5. Antibiotic Susceptibility Test

The antibiotic susceptibility of *Salmonella* strains was evaluated using the Kirby–Bauer disk diffusion test on Mueller–Hinton agar (MH, Becton Dickinson, Franklin Lakes, NJ, USA) [26]. In brief, bacterial cells cultivated in LB broth overnight were adjusted to an OD_600_ of 0.6–0.7, which is equivalent to 5 × 10^7^ colony-forming units (CFU)/mL. The bacterial suspension was spread on MH agar using sterile swabs, and antibiotic disks (BBL™ Sensi-Disc™, Becton Dickinson) were placed on the agar plates. After incubation at 37°C, the diameters of the growth inhibition zones were measured to determine antibiotic susceptibility. The tested antibiotic disks included 10 different antibiotics, as follows: amoxicillin/clavulanic acid (AMC, 30 μg), ampicillin (AM, 10 μg), cephalothin (CF, 30 μg), gentamicin (GM, 10 μg), kanamycin (K, 30 μg), nalidixic acid (NA, 30 μg), neomycin (N, 30 μg), ampicillin/sulbactam (SAM, 20 μg), sulfamethoxazole/trimethoprim (SXT, 25 μg), and tetracycline (TE, 30 μg). 

### 2.6. Animal Ethics

Animal experiments were approved by the Institutional Animal Care and Use Committee of Kangwon National University (approval No. KW-160201-1) and carried out in accordance with international law and policy guidelines, including the Guide for the Care and Use of Laboratory Animals (The National Academies Press, 8th edition, Washington, DC, USA).

### 2.7. Determination of Bacterial Virulence in Mice

Bacterial virulence was evaluated in vivo using the half-maximal lethal dose (LD_50_) estimation [23,27]. Six-week-old female BALB/c mice were purchased from Orient Bio Inc. (Seongnam, Korea) and acclimated for 7 days prior to infection. Mice were intraperitoneally (i.p.) injected with serially diluted *Salmonella* cells from 10^2^ to 10^7^ CFU/mouse, and the number of dead mice was recorded for 14 days. Moribund mice were humanely euthanized under the guidance of the Institutional Animal Care and Use Committee of Kangwon National University. LD_50_ values were calculated using the formula of log_10_ [50% end point] = A + (B × C), where A = log_10_ [infectious dose showing a mortality next below 50%], B = difference of logarithms = [50% − (mortality at infectious dose next below 50%)]/[(mortality next above 50%) − (mortality next below 50%)], and C = log_10_ [difference between serial infectious doses used in challenge studies].

To predict the bacterial burden in the infected animals, 7-week-old female BALB/c mice were injected i.p. with WT (10^3^ CFU/dose) and mutants (10^4^ CFU/dose) *Salmonella* strains and sacrificed to harvest the spleen at 7 days post-infection (dpi). The body weight and spleen weight were measured. Subsequently, the spleens of the infected mice were homogenized using TissueLyser II (Qiagen, Germantown, MD, USA) at 30 Hz for 1 min. The lysate aliquot was serially diluted and plated on *Salmonella–Shigella* (SS) agar (Difco, Becton Dickinson) to count the colonized cells.

### 2.8. Mouse Immunization and Challenge Experiments

Groups of 6-week-old female BALB/c mice (six mice per group) were immunized with *Salmonella* mutant strains (10^4^ CFU/dose) or the WT ST1120 strain (10^3^ CFU/dose). A group of negative-control mice was administered (i.p.) an equivalent volume of PBS. Mouse survival and weight were monitored for 56 days and the serum was collected at 0, 7, 14, 28, and 35 dpi. On day 21, all mice were orally challenged with the ST2173 strain at 10^8^ CFU/dose for the protection test and 10^10^ CFU/dose for the survival test. In the mouse protection test, challenged mice were sacrificed at 35 days after immunization (14 days after the challenge), and the liver and spleen were harvested to search for signs of hepatosplenomegaly and determine the bacterial load in these organs. The homogenized tissue lysates were plated on SS agar and the number of live *Salmonella* was counted. For the mouse survival test, the challenged mice were monitored for an additional 35 days after the challenges and the survival rate was recorded.

### 2.9. Enzyme-Linked Immunosorbent Assay (ELISA) Specific to S. Typhimurium OMPs

The specific antibody responses of IgG, IgM, and IgG subclasses (IgG1 and IgG2a) to *S*. Typhimurium OMPs were assessed using ELISA, as detailed previously [23,28]. Using triplicate sera from immunized and non-immunized mice, the optimal concentrations of serum and enzyme conjugates were determined based on the method of the checkerboard test [29,30]. The wells of microplates (Thermo Scientific Inc.) were coated with *Salmonella* OMPs by incubating OMPs at 0.5  μg/100 μL overnight at 4 °C, then blocked with 1% bovine serum albumin in PBS containing Tween 20 (PBS-T) at 37 °C for 2 h. After three washes with PBS-T, the wells were treated with the sera diluted at a 1:200 ratio. The plates were incubated at 37 °C for 1 h and then washed with PBS-T three times to remove antibodies with nonspecific binding to OMPs. Subsequently, the wells were treated with horseradish peroxidase (HRP)-conjugated antibodies, including the goat anti-mouse IgG (1:30000 dilution, Bethyl Laboratories, Montgomery, TX, USA), anti-mouse IgM (1:35000 dilution, Bethyl Laboratories), anti-mouse IgG1 (1:25000 dilution, Bethyl Laboratories), and anti-mouse IgG2a (1:30000 dilution, Bethyl Laboratories) secondary antibodies. After washing with PBS-T three times, 3,3′,5,5′-tetramethylbenzidine (TMB, Surmodics Inc., Eden Prairie, MN, USA) was added to each well as a chromogenic substrate and the enzymatic reaction was quenched by adding 0.5 M H_2_SO_4_ after 5 min of incubation. Colorimetric changes were measured at OD_450_ using an Epoch plate reader (BioTek, Suwanee, GA, USA).

### 2.10. Statistical Analysis

At least triplicates from independent experiments were used to interpret the results. All values were displayed as the mean ± standard deviation (SD). The significance of differences was set at *p* < 0.05. *p*-values were computed using one- or two-way analyses of variance with Tukey’s Multiple Comparison Test. GraphPad Prism 5 (GraphPad Software Inc., San Diego, CA, USA) was used for all statistical analyses.

## 3. Results

### 3.1. Attenuated Virulence of ΔfruR, ΔssrAB, and Δhfq Mutants in Mice Infection

**Figure 1:**
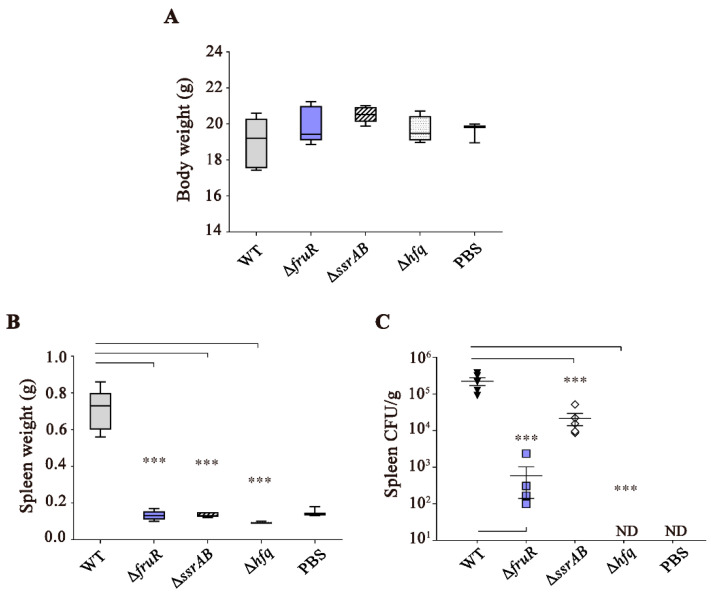
Bacterial virulence of the Δ*fruR*, Δ*ssrAB*, and Δ*hfq* mutant strains in mice. Groups of 7-week-old female BALB/c mice (five mice per group) were injected intraperitoneally with the ST mutant strains (Δ*fruR*, Δ*ssrAB*, or Δ*hfq*) at 10^4^ CFU/dose or with the wild-type (WT) strain at 10^3^ CFU/dose. At day 7 after infection, all mice were sacrificed and the body weight was measured (**A**). The spleens were isolated and weighed (**B**). Splenomegaly was not observed in three mutants of Δ*fruR*, Δ*ssrAB*, and Δ*hfq*. Homogenized spleen tissues were diluted using PBS and plated onto SS agar plates to calculate the bacterial number (**C**). Bacterial numbers were significantly low in the infection with Δ*fruR* or Δ*hfq*. Statistical analysis was performed using one-way ANOVA with Tukey’s multiple comparison test in compared with WT strain. Asterisks (***) indicate a significant difference (*p* < 0.001).

To induce substantial alterations in the physiological behavior of *Salmonella*, three global regulator genes encoding FruR, SsrAB, and Hfq were deleted individually in *Salmonella* Typhimurium strain 1120 (ST1120), which was isolated from swine feces [19]. The virulence of regulatory mutants was evaluated by LD_50_ estimation during BALB/c mouse infection. In comparison with WT *Salmonella* (LD_50_ = 10^3.1^), all three mutants exhibited a significantly attenuated virulence, with an increase in LD_50_ of at least 146-fold in mice infected with the Δ*fruR* mutant (Table 1). In murine salmonellosis, invasive *Salmonella* penetrates the intestinal epithelial barrier and spreads to deeper tissues, such as the spleen and liver, causing systemic infection. Therefore, *Salmonella*-induced splenomegaly is a primary pathological alteration observed during the process of *Salmonella* systemic infection [31,32]. We compared the development of splenomegaly between WT and mutant *Salmonella* strains by weighing the spleens harvested from infected mice. At day 7 after *Salmonella* infection, the body weights of infected mice were comparable between the WT and mutant strains, despite the use of different doses (WT: 10^3^ CFU/mouse vs. mutant: 10^4^ CFU/mouse; Figure 1A). As expected, the spleens of mice infected with WT *Salmonella* were enlarged, showing an approximately 4.7-fold increase in size. However, the three mutants (Δ*fruR*, Δ*ssrAB*, and Δ*hfq*) scarcely caused splenomegaly, even at a 10-fold higher dose (Figure 1B). To examine bacterial growth in the spleen tissues, the infected organ was homogenized and plated onto selective agar, and *Salmonella* colonies were counted (Figure 1C). The bacterial loads in the spleen were remarkably low in the mice infected with the Δ*fruR* (5.9 × 10^2^ CFU/g of spleen) and Δ*hfq* (not detected) mutants compared with those infected with the WT strain (2.2 × 10^5^ CFU/g of spleen). The Δ*ssrAB* mutant strain exhibited a 10-fold reduction in the bacterial load of the spleens (2.2 × 10^4^ CFU/g of spleen).

### 3.2. The ΔfruR, ΔssrAB, and Δhfq Mutants Are Defective in Survival Inside Macrophages

**Figure 2:**
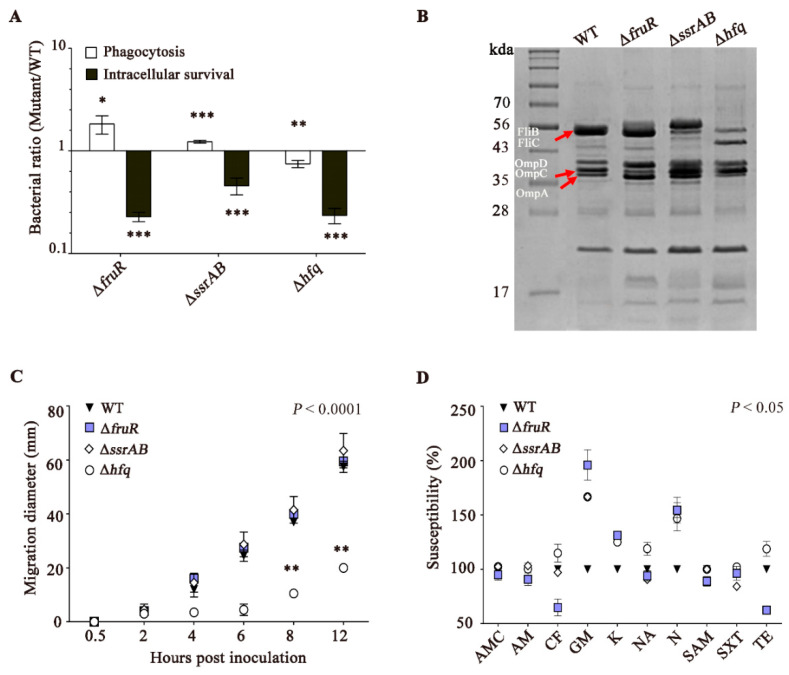
Characterization of the Δ*fruR*, Δ*ssrAB*, and Δ*hfq* mutant strains in vivo and in vitro. (**A**) Intracellular survival of the ST wild-type (WT) and mutant (Δ*fruR*, Δ*ssrAB*, and Δ*hfq*) strains in RAW264.7 murine macrophage cells. RAW264.7 cells were infected with each *Salmonella* strain at an MOI of 100 and the number of intracellular bacteria was counted at 2 h (phagocytosis) and 10 h (survival) post-infection. The ratio of the number of mutant/WT bacterial cells is presented (* *p* < 0.05; ** *p* < 0.01; *** *p* < 0.001). All three mutants showed attenuated intracellular survival at 10 h post-infection. (**B**) OMPs from WT and mutant strains were isolated and analyzed using 12% SDS–PAGE. Representative protein bands with altered production levels in comparison with WT were indicated with arrows. (**C**) Bacterial motility was compared between WT and mutant strains using semi-solid agar. The migration diameter (mm) of each bacterial strain was measured at 0.5, 2, 4, 6, 8, and 12 h, and analyzed by two-way analysis of variance (** *p* < 0.01). Bacterial motility was significantly impaired in the absence of Hfq. (**D**) The bacterial resistance to 10 different antibiotics was compared between the WT and three mutant strains using the Kirby–Bauer disk diffusion assay. The diameter of the growth inhibition zone was measured to determine antibiotic susceptibility, and the susceptibility of WT *Salmonella* to each antibiotic was set as 100%. In general, the three mutants were more susceptible to gentamicin, kanamycin, and neomycin, all of which are aminoglycoside antibiotics. The antibiotics tested included amoxicillin/clavulanic acid (AMC, 30 μg), ampicillin (AM, 10 μg), cephalothin (CF, 30 μg), gentamicin (GM, 10 μg), kanamycin (K, 30 μg), nalidixic acid (NA, 30 μg), neomycin (N, 30 μg), ampicillin/sulbactam (SAM, 20 μg), sulfamethoxazole/trimethoprim (SXT, 25 μg), and tetracycline (TE, 30 μg). The values are the averaged susceptibilities from triplicates.

To gain insight into the mechanisms underlying the attenuated virulence of these three mutants in mice, the intracellular survival ability was compared using murine macrophage cells. The growth of the Δ*fruR*, Δ*ssrAB*, and Δ*hfq* mutant strains was comparable to that of WT *Salmonella* in LB medium broth in vitro (Figure S1). However, all three mutants were significantly attenuated regarding intracellular survival at 10 h post-infection (Figure 2A), which was likely attributable to decreased SPI-2 expression [20]. Moreover, multiple structural devices expressed at the outer membrane determine the fate of *Salmonella* during host–pathogen interactions. The profiles of OMPs were compared between the WT and mutant strains (Figure 2B). Multiple proteins were differentially expressed in the absence of FruR, SsrAB, or Hfq. For example, the Δ*hfq* mutant strain produced less flagellin proteins, including FljB (52.5 kDa) and FliC (51.6 kDa), whereas the Δ*ssrAB* mutant strain reversed the flagellin type from FliC to FljB, which is probably attributable to flagellar-phase variation. In accordance with the decreased flagellin production detected in the Δ*hfq* mutant strain, bacterial motility was significantly impaired in the absence of Hfq (Figure 2C). In addition, protein bands ranging in size from 35 to 43 kDa showed different intensities between strains. The Δ*fruR* and Δ*ssrAB* strains in particular increased the production of porin proteins, such as OmpA (37.5 kDa), OmpC (41.2 kDa), and OmpD (41.3 kDa). The altered production of porin proteins in these mutants might compromise the membrane integrity and affect bacterial resistance against antimicrobials [33,34]. To test this possibility, bacterial strains were treated with 10 different antibiotics and the susceptibility to these agents was compared (Figure 2D). In comparison with WT *Salmonella*, the three mutants were more susceptible to gentamicin, kanamycin, and neomycin, all of which are aminoglycoside antibiotics. A biochemical test using the API kit showed that the Δ*fruR*, Δ*ssrAB*, and Δ*hfq* mutant strains shut down biochemical activities associated with arginine dihydrolase, citrate utilization, and lysine decarboxylase, while promoting amygdalin and sucrose fermentation activities, indicating altered metabolic pathways associated with these compounds in these mutant strains (Table S3).

Taken together, these results suggest that the three regulators of FruR, SsrAB, and Hfq modulate a variety of cellular activities, including virulence, motility, antibiotic resistance, and metabolism.

### 3.3. Evaluation of the Immune Responses Induced by the ΔfruR, ΔssrAB, and Δhfq Mutants

**Figure 3:**
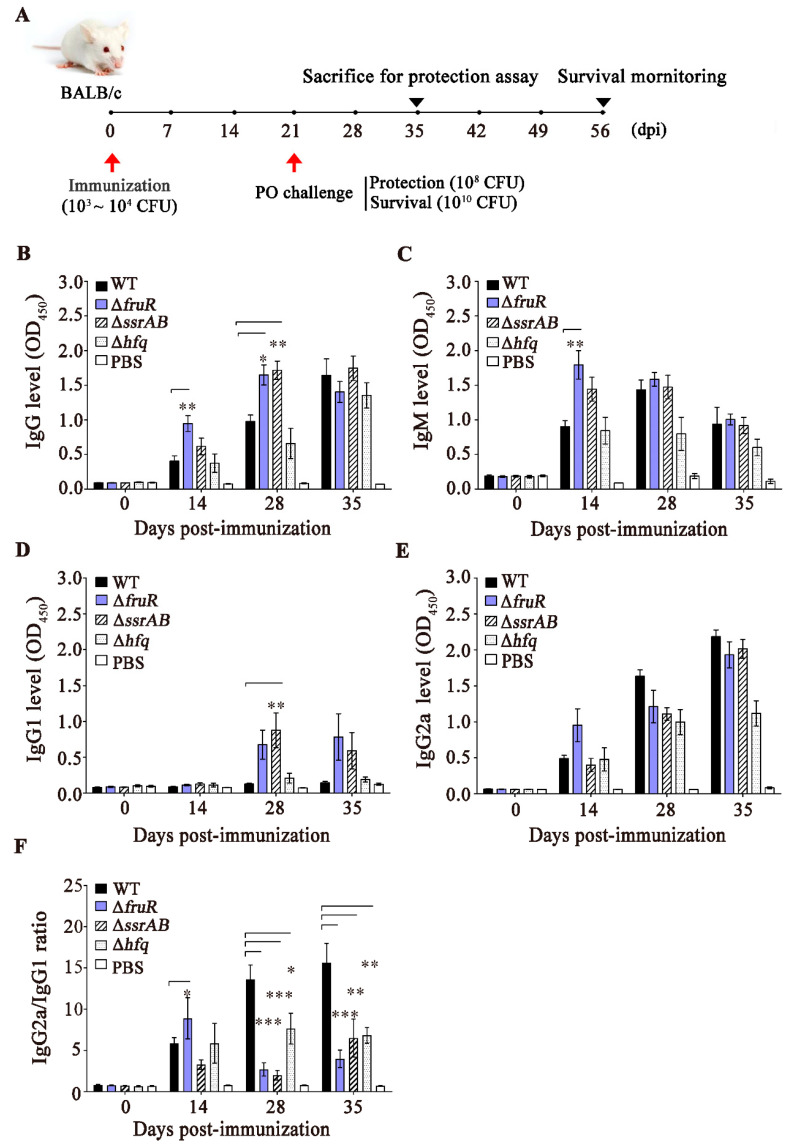
Stimulation of *Salmonella*-specific antibodies in immunized mice. Groups of six BALB/c mice were inoculated intraperitoneally with the three mutant strains (10^4^ CFU/mouse), the wild-type (WT) strain (10^3^ CFU/mouse), or PBS (100 μL/mouse; N control). (**A**) At 21 days post-infection (dpi), all mice but the N control mice were orally challenged with the virulent ST2173 strain at a dose of LD_50_ (10^8^ CFU/mouse). Positive (P) control mice were pre-inoculated with PBS similar to the N control and then challenged with ST2137 at the same dose. The antibody levels of (**B**) IgG, (**C**) IgM, and (**D** and **E**) IgG subclasses, including IgG1 and IgG2a, were measured using the sera collected from the infected mice at 0, 14, 28, and 35 dpi. Data pertaining to IgG, IgM, and IgG subclass levels are shown as the mean ± SEM. In comparison with the WT, the Δ*fruR* mutant caused rapid increments in IgG and IgM levels at 14 dpi, while the Δ*ssrAB* mutant strain increased IgG levels at 28 dpi. (**F**) The levels of IgG1 and IgG2a are displayed as a ratio (IgG2a/IgG1). Immunization with the Δ*fruR* and Δ*ssrAB* mutant strains led to the lower IgG2a/IgG1 ratios. Statistical analysis of the IgG, IgM, and IgG subclass levels was conducted using one-way ANOVA with Tukey’s multiple comparison test in comparison with WT strain (* *p* < 0.05; ** *p* < 0.01; *** *p* < 0.001).

The immunogenicity of the three mutant strains was compared by measuring the levels of serum IgG, IgM, and IgG subclasses (IgG1 and IgG2a) after immunization of female BALB/c mice. Groups of mice were immunized (i.p.) with the WT and mutant strains and then orally infected with a WT *S.* Typhimurium strain (ST2173) at 21 dpi (Figure 3A). Mouse sera were collected on a weekly basis and to measure the levels of *Salmonella*-specific antibodies. The Δ*fruR* mutant caused rapid increments in IgG and IgM levels at 14 dpi compared with the WT and Δ*hfq* mutant strains, whereas the Δ*ssrAB* mutant strain remarkably increased IgG levels at 28 dpi (Figure 3B,C). However, the levels of IgG and IgM were comparable among strains at 35 dpi. The IgG2a/IgG1 ratio is indicative of the type of immune responses triggered by T-helper 1 (Th1) or Th2 effector cells. Mice immunized with the WT and Δ*hfq* mutant strains did not exhibit significant changes in IgG1 levels up to 35 dpi, whereas immunization with the Δ*fruR* and Δ*ssrAB* mutant strains caused surges in IgG1 levels at 28 and 35 dpi, respectively (Figure 3D). In contrast with the IgG1 responses, the IgG2a levels increased gradually up to 35 dpi, regardless of the *Salmonella* strains used in the immunization, although the Δ*hfq* mutant strain stimulated IgG2a production to a lesser extent than did the remaining three strains (Figure 3E). Notably, the vigorous IgG2a production triggered by either the WT or mutant strains indicates that immunization with *Salmonella* strains could elicit a Th1-mediated immune response proficiently. However, the lower IgG2a/IgG1 ratios afforded by the Δ*fruR* (ranging from 2.7 at 28 dpi to 8.9 at 14 dpi) and Δ*ssrAB* (ranging from 2.0 at 28 dpi to 6.5 at 35 dpi) mutants compared with WT *Salmonella* (ranging from 5.8 at 14 dpi to 15.7 at 35 dpi) suggest that the Δ*fruR* and Δ*ssrAB* mutant strains were more competent in stimulating an IgG1-associated Th2 immune response as well as an IgG2a-associated Th1 immune response than was the WT *Salmonella* strain (Figure 3F).

### 3.4. Prophylactic Effect of the ΔfruR Mutant against Virulent Salmonella Infection in Mice

**Figure 4:**
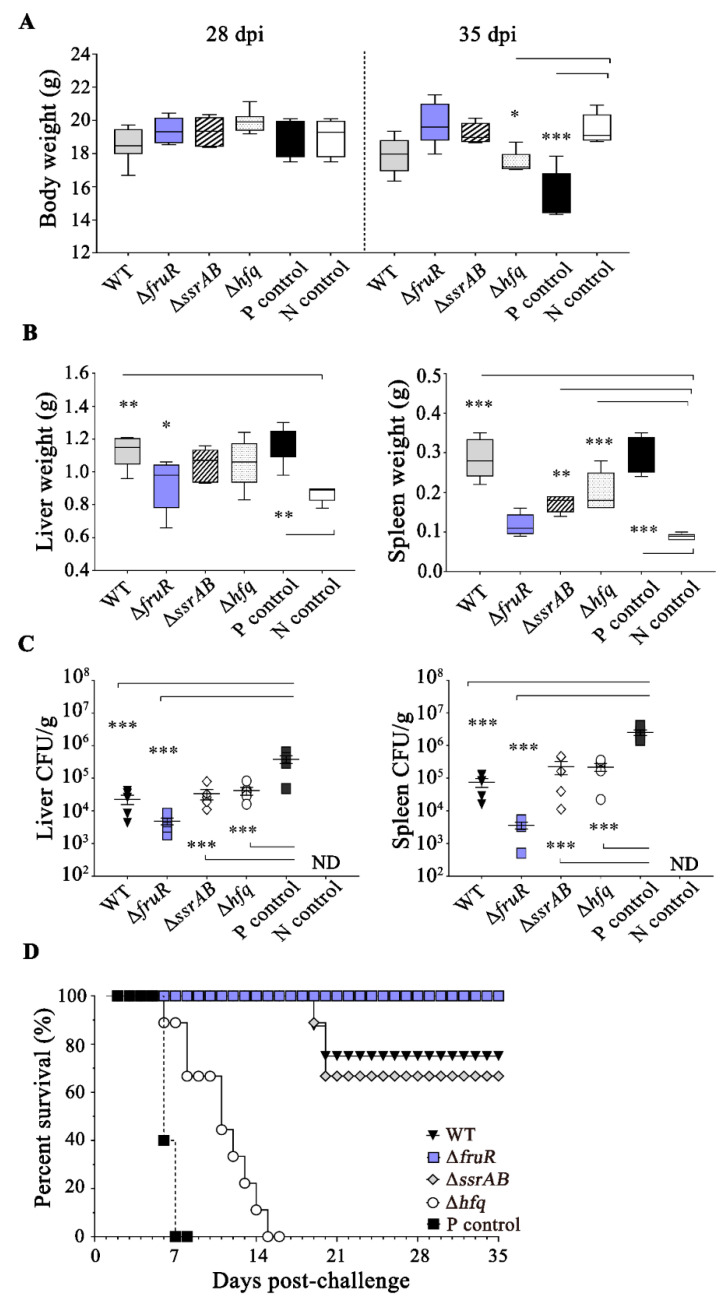
Comparison of prophylactic effects between *Salmonella* mutant strains in mice. As depicted in Figure 3A, groups of mice were immunized with wild-type (WT) or mutant *Salmonella* strains and then challenged with the ST2173 strain (10^8^ CFU/mouse for the protection assay; 10^10^ CFU/mouse for the survival assay). The N control received PBS in both inoculations, whereas the P control received ST2173 inoculation at challenge tests after PBS administration. The body weight was measured at 28 and 35 dpi (**A**). All mice tested in the protection assay were euthanized at 14 days post-challenge (14 dpc; 35 dpi), and the livers and spleens were weighed (**B**) and used to count the ST2173 cells residing in these organs (**C**). Mice immunized with the Δ*fruR* mutant strain exhibited no signs of hepatosplenomegaly, which was contrast to the cases of WT, Δ*ssrAB* and Δ*hfq* mutant strains showing enlarged livers and spleens. Accordantly, immunization with the Δ*fruR* mutant strain resulted in the lowest bacterial burden in the organ. Statistical analysis was performed using one-way ANOVA with Tukey’s multiple comparison test. The asterisks indicate significant differences as follows: * *p* < 0.05; ** *p* < 0.01; *** *p* < 0.001. In the survival assay, mice challenged with ST2173 were further monitored up to 35 dpc (56 dpi) and their survival rates plotted (**D**). Mice immunized with the Δ*fruR* mutant strain exhibited a survival of 100% against lethal ST2173 infection.

The prophylactic efficacy of pre-infection with the three mutant strains was investigated in BALB/c mice by challenging them with virulent *Salmonella* (ST2173) infection at 21 dpi (Figure 3A). Mice that were pre-infected with *Salmonella* strains or PBS showed comparable body weights up to 28 dpi; however, additional ST2173 infection at a dose of LD_50_ caused alterations in body weight that were dependent on the pre-infection composition (Figure 4A). Mice that were not immunized with any of the *Salmonella* strains but were administered the virulent ST2173 strain (P control) lost 23% of their weight compared with healthy mice (N control, administered PBS alone) at 35 dpi (14 days post-challenge; 14 dpc). However, mice that were pre-infected with the *Salmonella* strains, with the exception of the Δ*hfq* mutant, did not lose weight, even after lethal ST2173 infection. Notably, mice that were immunized with the Δ*fruR* mutant gained weight for 14 days after ST2173 infection, showing body weights equivalent to those of the N control (Figure 4A). Pathological signs such as hepatomegaly and splenomegaly are typically observed in mice suffering from systemic infection with *Salmonella*, as shown in the P control (Figs 4B and S2). The liver and spleen also became enlarged in the challenged mice after pre-infection with WT or Δ*ssrAB* and Δ*hfq* mutant strains (Figure 4B). By contrast, mice immunized with the Δ*fruR* mutant strain exhibited no signs of hepatosplenomegaly, showing liver and spleen weights that were similar to those of healthy mice (Figure 4B). The bacterial burden of the liver and spleen exhibited a tendency that was reminiscent of the phases of hepatosplenomegaly. Moreover, immunization with the Δ*fruR* mutant strain resulted in the lowest bacterial burden in the organ among the tested *Salmonella* strains, showing a 78.9-fold (liver) and 692.7-fold (spleen) lower number of *Salmonella* compared with the P control challenged without immunization (Figure 4C). Immunization with the Δ*ssrAB* and Δ*hfq* mutant strains moderately reduced the bacterial counts in the liver and spleen compared with the P control cases. The monitoring of mouse survival for 35 days after the challenge with ST2173 revealed that P control mice not immunized with any *Salmonella* strains showed severe morbidity, including a ruffled hair coat, dull eyes, shivering, and weight loss, and all succumbed to death within 7 dpc. However, mice immunized with the Δ*fruR* mutant strain exhibited a survival of 100% against lethal ST2173 infection (Figure 4D), indicating the potency of the Δ*fruR* mutant strain as a live vaccine. Interestingly, WT *Salmonella* and the Δ*ssrAB* mutant displayed substantial protection activity (75% survival with WT *Salmonella*; 66.7% survival with the Δ*ssrAB* strain) against ST2173 infection (Figure 4D). Taken together, these findings suggest that the Δ*fruR* mutant strain, as a potential candidate vaccine, afforded a successful protective activity against *Salmonella* infection.

## 4. Discussion

NTS strains commonly cause self-limiting illness with a low lethal rate [35]. However, certain NTS serovars, including *S.* Typhimurium, *S.* Enteritids, and *S.* Dublin, are occasionally invasive and disseminate to deeper tissues, causing deadly diseases such as septicemia and meningitis [36,37]. Furthermore, some highly invasive strains, such as *S.* Typhimurium ST 313 and ST 34, are resistant to multiple antibiotics recommended as the first-line treatment for these infections [37,38]. According to the Global Burden of Disease (GBD) Study, the burden of invasive NTS diseases was estimated to be 535,000 cases, including 77,500 deaths in 2017, with the most vulnerable populations being young children, the elderly, and immunocompromised individuals [39]. High case fatality rates and emerging multidrug resistance underscore the urgent need to develop proficient vaccines against invasive NTS infection. Currently, although there is no licensed vaccine against invasive NTS, several vaccine candidates are under clinical field trials, including live attenuated vaccines, protein–polysaccharide vaccines (glycoconjugate vaccines), protein vaccines, and Outer Membrane Vesicle (OMV) -based vaccines [10]. Regarding toxicity, immunogenicity, longevity, immunotherapeutic spectrum, and production cost, each vaccine strategy has advantages and disadvantages. Live attenuated vaccines deliver a wide repertoire of antigens on a temporal and spatial basis in the interaction with the host immune system and are expected to elicit successfully both cellular and humoral responses [40]. The cost-effectiveness of both the production and administration of vaccines is also a considerable advantage of live attenuated vaccines. However, live attenuated vaccines, which are generally constructed by deleting genes that are essential for virulence and proliferation in vivo, have the possibility of genetic reversion to a WT phenotype via the re-acquisition of the deleted genes, thus imposing a safety risk. Of course, this possibility can be debilitated by mutating multiple loci localized separately in the genome [41]. In addition, live attenuated vaccines are prone to be excreted in the stool over a long period. For example, the WT05 candidate, which is defective in aromatic acid biosynthesis and SPI-2-associated virulence because of the lack of *aroC* and *ssaV*, respectively, failed at the clinical stage because of long-lasting fecal shedding (for more than 3 weeks) [42]. Prolonged stool excretion is problematic considering the possibility of *Salmonella* transmission through food and water contamination. To date, many *Salmonella* strains have been suggested as candidates for live attenuated vaccines against invasive NTS; *Salmonella* strains devoid of global transcriptional regulators such as PhoP [43], strains defective in signal transduction via small signaling molecules such as ppGpp [44], and strains unable to biosynthesize aromatic compounds and purines [45] In spite of the protection activity in animal models, the superiority as a live attenuated vaccine in human model cannot be guaranteed unless the dynamic interaction between vaccines and host immune system is fully understood. Unfortunately, there is no live vaccine applicable to humans yet. 

In an effort to develop a potent live attenuated vaccine against invasive NTS, we compared three *S.* Typhimurium mutant strains, i.e., Δ*fruR*, Δ*ssrAB*, and Δ*hfq*, in regard to virulence, immunogenicity, and prophylactic activity. The three genes of *fruR*, *ssrAB*, and *hfq* encode transcriptional or post-transcriptional regulators, and their deletions are expected to modulate the expression of multiple genes directly or indirectly, leading to extensive alterations in bacterial behavior and physiology, such as decreased SPI-2 expression in an acidic minimal medium mimicking the intracellular environment [20]. *Salmonella* lacking Hfq exhibited a marked attenuation of virulence during mouse infection and cleared out from the spleen at 7 dpi, yielding decreases in intracellular survival and motility; however, its ability to stimulate an immune response was marginal, indicating that the Δ*hfq* mutant strain failed to protect mice against lethal *Salmonella* infection. Conversely, the Δ*fruR* and Δ*ssrAB* strains exhibited a reduced virulence, but exerted sufficient immunogenicity providing a protection efficacy of 100% (Δ*fruR*) and 75% (Δ*ssrAB*) survival against infection with virulent *Salmonella*. Considering the role of SsrAB as the master regulators of SPI-2 and its associated genes, the attenuated virulence of the Δ*ssrAB* mutant strain was likely attributable to a malfunctioning type 3 secretion system (T3SS) encoded by SPI-2. The temporal and spatial translocation of virulence effectors via T3SS during host infection is critical for *Salmonella* to colonize and persist successfully inside the host animals [46]. FruR, which was initially known as the transcriptional repressor of the *fru* operon, is a dual regulator with both activation and repression activities that is implicated in the regulation of a large number of genes associated with carbon metabolism [17]. Because of these multifaceted roles of FruR in carbon metabolism, it is also termed catabolite repressor/activator (Cra). In pathogenic Gammaproteobacteria, including *Salmonella enterica*, *Shigella flexneri*, and enterohemorrhagic *Escherichia coli*, the lack of FruR also impairs bacterial virulence during host infection [20,47,48]. Carbohydrates are not only incorporated in energy metabolism pathways, but also exploited as structural building blocks, such as d-glucosamine-6-phosphate for bacterial peptidoglycan, lipopolysaccharide (LPS), and biofilm production. Therefore, the ability to maintain carbon homeostasis in response to extracellular environments is important for bacterial growth and resistance. *Salmonella* devoid of FruR might be incompetent in proliferation in vivo because of an imbalanced carbon flow; moreover, *Salmonella* may also undergo structural alterations in peptidoglycan and LPS compositions. However, despite the plausible defects that occur in the absence of FruR, the Δ*fruR* mutant strain, as a prophylactic vaccine, was able to stimulate IgG and IgM production more promptly and strongly than were the WT and Δ*ssrAB* mutant strains, and afforded the best protection capacity among the tested strains against lethal *Salmonella* infection.

The innate immune system, which harnesses macrophages and neutrophils, is the primary line of defense against *Salmonella* infection in the gut; however, subsequently, the adaptive immune system plays a greater role in eradicating *Salmonella* by providing effective protection to subsequent challenges with related *Salmonella* groups [16]. In this context, the importance of CD4^+^ Th-cell-mediated immunity has gained attention [10]. Naïve CD4^+^ T cells are activated into the two subsets of Th1 and Th2 cells depending on the repertoires of cytokines produced by these Th cells. Th1 cells secrete IL-2, IFN-γ, and TNF-α and stimulate cell-mediated immunity and inflammation, whereas Th2 cells secrete IL-4, IL-5, and IL-13 and promote humoral immunity via B-cell activation and differentiation. Given that invasive NTS exploits host cells (even macrophages) to evade various immune responses, both humoral and cell-mediated immune responses are required for proficient protection against fatal *Salmonella* infection. The ratio of IgG2a/IgG1 is typically used as a surrogate marker to predict the type of immune responses dominated by Th1 or Th2 cells. The Δ*fruR* and Δ*ssrAB* mutants showed lower IgG2a/IgG1 ratios than did WT *Salmonella*, despite the comparable IgG2a levels among them, indicating that these two mutant strains can successfully induce Th2-cell-mediated humoral immunity as well as Th1-cell-mediated cellular immunity. Accordingly, immunization with these two mutants provided substantial protection against lethal doses of *Salmonella* infection. In consideration of the inevitable drawback of live attenuated vaccines, i.e., the genetic reversion to a WT phenotype, engineering *Salmonella* devoid of both FruR and SsrAB may represent a promising approach to the development of a feasible vaccine.

## 5. Conclusions

In order to develop a live attenuated vaccine against invasive NTS, three *S.* Typhimurium mutant strains lacking FruR, SsrAB, or Hfq were evaluated for virulence, immunogenicity, and prophylactic activity. All three mutants were attenuated in virulence. However, immunization with Δ*hfq* mutant strain rarely stimulated the host immune response and failed to protect mice against *Salmonella* infection. On the other hand, the Δ*fruR* and Δ*ssrAB* strains exerted sufficient immunogenicity in mice and showed remarkable prophylactic efficacy against lethal *Salmonella* infection. With regard to antibody production, Δ*fruR* mutant stimulated IgG and IgM production more rapidly and strongly than Δ*ssrAB* mutant. The Δ*fruR* and Δ*ssrAB* mutant strains are promising vaccine candidates against invasive NTS infection and deserve further exploration for developing a live attenuated NTS vaccine.

## Figures and Tables

**Table 1:** LD_50_ values of *S.* Typhimurium strains in BALB/c mice.

Strains	Infection Dose (CFU/Mouse)	Number of Dead Mice/ Total Mice Number	LD_50_ (CFU/Dose)
Wild-type ST1120	10^5^	3/4	10^3.1^
	10^4^	3/4	
	10^3^	0/4	
	10^2^	0/4	
Δ*fruR*	10^7^	4/4	10^5.3^
	10^6^	4/4	
	10^5^	0/4	
	10^4^	0/4	
Δ*ssrAB*	10^7^	3/4	10^6.5^
	10^6^	1/4	
	10^5^	0/4	
	10^4^	0/4	
Δ*hfq*	10^7^	4/4	10^6.5^
	10^6^	0/4	
	10^5^	0/4	
	10^4^	0/4	

## Data Availability

Not applicable.

## Supplementary Materials

The following are available online at https://www.mdpi.com/article/10.3390/vaccines10091413/s1, Table S1: Bacterial strains and plasmids used in this study. Table S2: Primers used in this study. Table S3: Analysis of the biochemical phenotypes of the *S.* Typhimurium Δ*fruR* mutant strain. Figure S1: Measurement of bacterial growth in the *ΔfruR* mutant in LB broth. Figure S2: The enlarged livers and spleens of mice immunized with each strain after challenge. Reference [49] is cited in supplementary materials.
