# Supplementary material for: Evaluation of Salmonella Typhimurium Lacking fruR, ssrAB, or hfq as a Prophylactic Vaccine against Salmonella Lethal Infection
Source: Vaccines (Basel). 2022 Aug 28;10(9):1413. doi: 10.3390/vaccines10091413 (PMC9506222; doi:10.3390/vaccines10091413)
Supplement: Supplementary file 1 [file vaccines-10-01413-s001.zip › vaccines-1828359-supplementary.pdf]

Supplement Table S1. Bacterial strains and plasmids used in this study

Strains	Relevant characteristics	References
ST1120	Wild-type *S.* Typhimurium isolated in Korea	[1]
ST2165	Δ*fruR*	[2]
ST2166	Δ*ssrAB*	[2]
ST2167	Δ*hfq*	[2]
ST14028	ATCC14028	ATCC
ST2173	ST14028 with plasmid pBBR1-MCS4 (AM^R)	[3]
**Plasmids**	**Characteristics**	
pCP20	FLP recombinase, temperature-sensitive replication (AM^R)	[4]
pBBR1-MCS4	Broad-host-range cloning vector (AM^R)	[5]

Supplement Table S2. Primers used in this study

**Primer**	**Sequence (5′ to 3′)**
*fruR*_DF	CATCGGTTGGGCTTTTTTTTG
*fruR*_DR	TGTCAAGCAAGTCACAACG
ssrAB-DF	TTGAAGCATCGTCTCCTG
*ssrAB*_DR	GGCGATTCTATCATTCGG
*hfq*_DF	CAGGTTTTGAAGCAGAAGTC
*hfq*_DR	TTCAACTGCCTTACCTTCAC

Supplement Table S3. Analysis of the biochemical phenotypes of the *S.* Typhimurium Δ*fruR* mutant strain

Biochemical phenotypes	Strain characteristics			
	**Wild-type**	**Δ*fruR***	**Δ*ssrAB***	**Δ*hfq***
Acetoin production	−	−	−	−
Amygdalin fermentation	−	**+**	**+**	**+**
Arabinose fermentation	+	+	+	+
Arginine dihydrolase	+	**−**	**−**	**−**
Citrate utilization	+	**−**	**−**	**−**
β-Galactosidase	−	−	−	−
Gelatinase	−	−	−	−
Glucose fermentation	+	+	+	+
H_2S production	+	+	+	+
Indole production	−	−	−	−
Inositol fermentation	+	+	+	+
Lysine decarboxylase	+	**−**	**−**	**−**
Mannitol fermentation	+	+	+	+
Melibiose fermentation	+	+	+	+
Ornithine decarboxylase	+	+	+	+
Rhamnose fermentation	+	+	+	+
Sorbitol fermentation	+	+	+	+
Sucrose fermentation	−	**+**	**+**	**+**
Tryptophan deaminase	−	−	−	−
Urease	−	−	−	−

Supplement Figure S1.

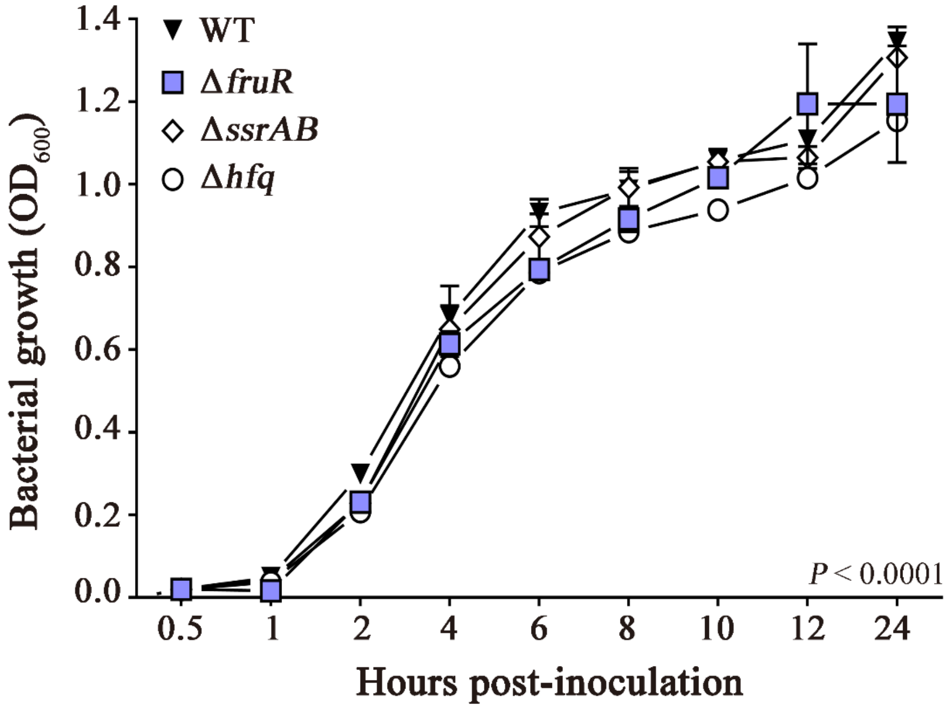

Supplementary Figure S1. Measurement of bacterial growth in the *ΔfruR* mutant in LB broth. Growth curve of ST mutant strains (*ΔfruR*, *ΔssrAB*, and *Δhfq*) and WT strains that were cultured in LB broth. The OD_{600} values indicated at each time point represent the average of three replicates.

Supplement Figure S2.

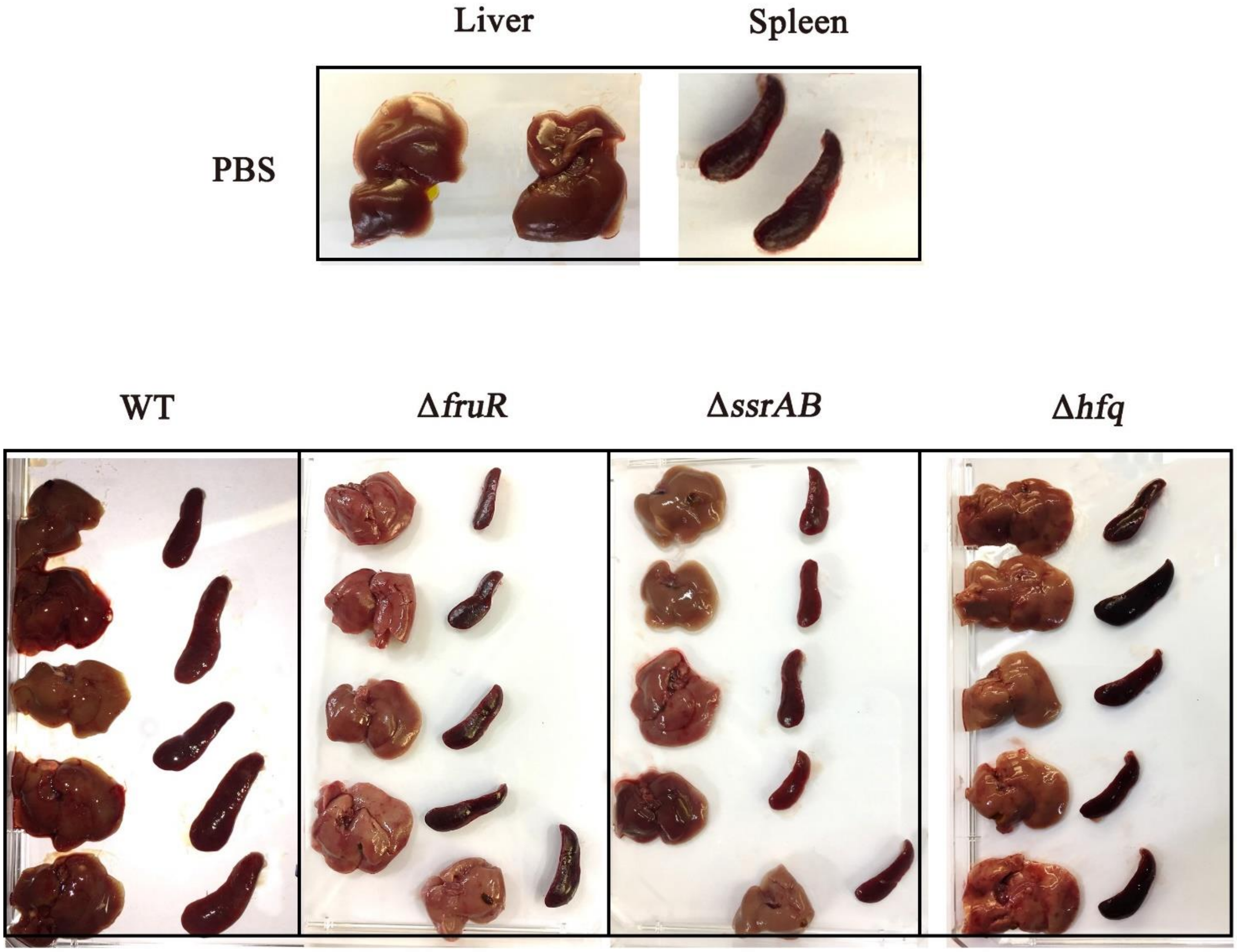

Supplementary Figure S2. The enlarged livers and spleens of mice immunized with each strain after challenge. The mice that were immunized with ST mutant strains (*ΔfruR*, *ΔssrAB*, or *Δhfq*) or WT strains were sacrificed and their organs (liver and spleen) were collected and observed. The PBS negative (N) control was not challenged.

References

1. Kim, S.; Kim, E.; Park, S.; Hahn, T. W.; Yoon, H. Genomic approaches for understanding the characteristics of *Salmonella enterica* subsp. enterica serovar Typhimurium ST1120, isolated from swine feces in Korea. *J. Microbiol. Biotechnol*. **2017**, 27, 1983–1993.

2. Yoon, H.; McDermott, J. E.; Porwollik, S.; McClelland, M.; Heffron, F. Coordinated regulation of virulence during systemic infection of *Salmonella enterica* serovar Typhimurium. *PLoS Pathog.* **2009**, 5, e1000306.

3. Park, S.; Jung, B.; Kim, E.; Hong, S. T.; Yoon, H.; Hahn, T. W. *Salmonella* Typhimurium lacking YjeK as a candidate live attenuated vaccine against invasive *Salmonella* infection. *Front Immunol.* **2020**, 11, 1277.

4. Datsenko, K.A.; Wanner, B.L. One-step inactivation of chromosomal genes in *Escherichia coli* K-12 using PCR products. *Proc. Natl. Acad. Sci. U S A.* **2000**, 97, 6640–6645.

5. Truong, Q.L.; Cho, Y.; Park, S.; Park, B. K.; Hahn, T. W. *Brucella abortus* mutants lacking ATP-binding cassette transporter proteins are highly attenuated in virulence and confer protective immunity against virulent *B. abortus* challenge in BALB/c mice. *Microb. Pathog.* **2016**, 95, 175–185.
